# Enhancing the Carrier Mobility and Bias Stability in Metal–Oxide Thin Film Transistors with Bilayer InSnO/a-InGaZnO Heterojunction Structure

**DOI:** 10.3390/mi15040512

**Published:** 2024-04-11

**Authors:** Xiaoming Huang, Chen Chen, Fei Sun, Xinlei Chen, Weizong Xu, Lin Li

**Affiliations:** 1 College of Integrated Circuit Science and Engineering, Nanjing University of Posts and Telecommunications, Nanjing 210023, China; 1021021012@njupt.edu.cn (C.C.); 1222228325@njupt.edu.cn (F.S.); 1023223211@njupt.edu.cn (X.C.); 2 National Laboratory of Solid State Microstructures, Nanjing University, Nanjing 210093, China; njuphyxwz@nju.edu.cn; 3 Key Laboratory of Laser Technology and Optoelectronic Functional Materials of Hainan Province, College of Physics and Electronic Engineering, Hainan Normal University, Haikou 571158, China; lin.li@hainnu.edu.cn

**Keywords:** ITO/a-IGZO heterojunction TFTs, conduction band offset, electrical performance, TCAD silvaco simulation

## Abstract

In this study, the electrical performance and bias stability of InSnO/a-InGaZnO (ITO/a-IGZO) heterojunction thin-film transistors (TFTs) are investigated. Compared to a-IGZO TFTs, the mobility (*µ_FE_*) and bias stability of ITO/a-IGZO heterojunction TFTs are enhanced. The band alignment of the ITO/a-IGZO heterojunction is analyzed by using X-ray photoelectron spectroscopy (XPS). A conduction band offset (∆*E_C_*) of 0.5 eV is observed in the ITO/a-IGZO heterojunction, resulting in electron accumulation in the formed potential well. Meanwhile, the ∆*E_C_* of the ITO/a-IGZO heterojunction can be modulated by nitrogen doping ITO (ITON), which can affect the carrier confinement and transport properties at the ITO/a-IGZO heterojunction interface. Moreover, the carrier concentration distribution at the ITO/a-IGZO heterointerface is extracted by means of TCAD silvaco 2018 simulation, which is beneficial for enhancing the electrical performance of ITO/a-IGZO heterojunction TFTs.

## 1. Introduction

Over the past decade, amorphous InGaZnO (a-IGZO) thin-film transistors (TFTs) have been intensively investigated as pixel switching devices for active-matrix display applications. The competitive superiority of a-IGZO TFTs is based on the fact that they can offer high carrier mobility, high optical transparency, and low off-state leakage compared with traditional Si-based TFTs [1,2,3], and their manufacturing cost is lower than that of low-temperature polycrystalline silicon (LTPS) TFTs with large-area uniformity [4,5]. However, due to the high density of subgap defects existing in the bandgap of a-IGZO, the carrier mobility (*μ_FE_*) and electrical stability are insufficient for advanced display applications such as AR/VR displays, flexible logic circuits, three-dimensional (3D) displays, and low-power mobile devices [6,7,8,9]. It has been reported that the subgap states mainly originate from oxygen vacancy-related defects induced by the structural disorder in a-IGZO [10], which affects the electrical properties and stability of TFTs by trapping electrons or holes in the channel layer and device interfacial region under gate bias stress, light illumination, and thermal stress [11]. To overcome this limitation of a-IGZO TFTs, the bandgap engineering of the bilayer heterojunction oxide channel has been proposed to enhance the electrical performance and bias stability of a-IGZO TFTs [12,13]. In the bilayer heterojunction structure, the improved electrical performance is caused by the large conduction band offset (∆*E_C_*) between two oxide channels, which will form the carrier confinement in the heterojunction potential well due to the bandgap pinning effects [14,15]. For example, it has been reported that the InZnO/a-IGZO, InSnO/a-IGZO, and InO/a-IGZO bilayer channels all exhibit a much higher *μ_FE_* than a-IGZO TFTs, which is attributed to the large difference in the Fermi energy levels of both oxide semiconductor layers. The electrons, which are the main carriers of most amorphous oxide semiconductors, are confined in the potential well constructed by the bandgap discrepancy, forming a quasi-two-dimensional electron gas (q2DEG) at the bilayer heterointerface [16,17,18,19]. In addition, it has been demonstrated that the electron trapping phenomenon could be suppressed by the formation of ∆*E_C_* between two oxide semiconductors, enhancing the bias stress stability of heterojunction TFTs [20,21]. Since the carrier confinement and trapping in the heterojunction structure depend on the ∆*E_C_* of both channel layers, an in-depth study of the effect of ∆*E_C_* on carrier confinement and transport properties of heterostructure is the key to the future optimization of the electrical characteristics of heterojunction TFTs. In this study, single-active-layer a-IGZO TFTs, bilayer ITO/a-IGZO heterojunction TFTs and ITON/a-IGZO heterojunction TFTs are fabricated, and the electrical properties and positive gate bias stability of TFTs are studied. It is found that the bilayer heterojunction TFTs exhibit improvement in *μ_FE_* and bias-stress stability compared with those of single-active-layer a-IGZO TFTs. The energy band alignment and band bending at the ITO/a-IGZO and ITON/a-IGZO heterojunction interface are revealed by X-ray photoelectron spectroscopy (XPS) and transmission spectra measurements. The conduction band offset (∆*E_C_*) is determined as 0.5 eV and 0.4 eV for the ITO/a-IGZO and ITON/a-IGZO heterojunction interface, respectively. The Δ*E_C_* is large enough to form an architecture containing both a potential well and barrier at the heterojunction interface, which agrees with the improvement in *μ_FE_* and bias-stress stability of the bilayer heterojunction TFTs. Meanwhile, it is found that the band alignment of the ITO/a-IGZO heterojunction could be modulated by N doping ITO. Lastly, the improved electrical performance of bilayer heterojunction TFTs is analyzed by TCAD simulations.

## 2. Device Fabrication and Simulation

**Figure 1:**
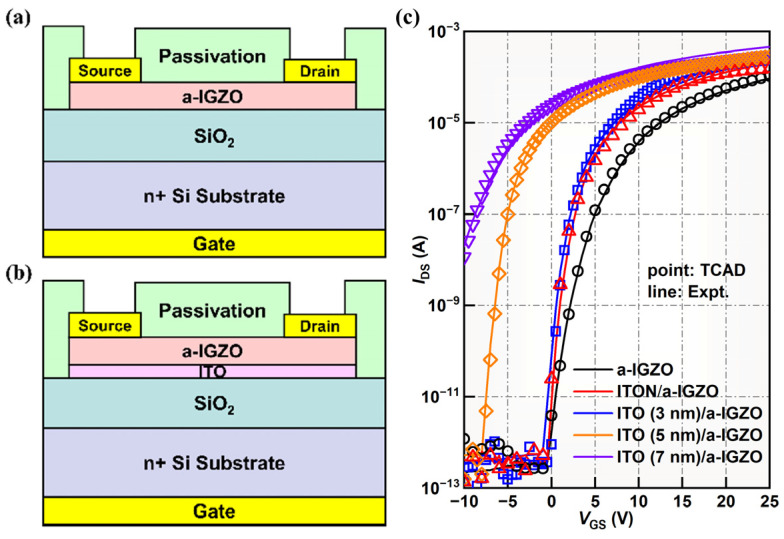
Schematic diagram for bottom-gate TFTs with (**a**) single-layer a-IGZO and (**b**) a ITO/a-IGZO heterojunction channel; (**c**) the transfer characteristics of the a-IGZO TFTs and ITO/a-IGZO TFTs with different ITO thicknesses. The measurement data (solid lines) fitted well with the TCAD simulation (circles).

In this work, the single-active-layer a-IGZO TFTs and bilayer ITO/a-IGZO heterojunction TFTs were fabricated on a heavily doped n-Si substrate with a staggered bottom-gate structure, as shown in Figure 1a,b. First, SiO_2_ (200 nm) was deposited by means of plasma-enhanced chemical vapor deposition (PECVD) at T = 300 °C. The bottom ITO channel layer (3, 5, and 7 nm) was then grown using a DC-sputtering system with a plasma power of 100 W and a mixture reactive gas ratio of Ar/O_2_ = 25%, and the top a-IGZO layer was continuously deposited without a vacuum break with a mixture reactive gas ratio of Ar/O_2_ = 20%. The composition of the ceramic target used had an atomic ratio of In:Ga:Zn = 2:2:1. The stacked ITO/a-IGZO channel layers were fixed at 40 nm. Meanwhile, to fabricate ITON/a-IGZO heterojunction TFTs, the bottom ITON channel layer was deposited using the DC-sputtering system in a mixture of Ar, O_2_ and N_2_ (Ar/O_2_/N_2_ = 30 sccm/6 sccm/4 sccm) using a target of In_2_O_3_:Sn_2_O_3_ = 90:10. Next, the devices’ active regions were formed by using photolithography and wet chemical etching. The Ti/Au (20/80 nm) bilayer film was deposited by means of electron-beam evaporation to form the drain/source (D/S) electrode, and a 100 nm thick SiO_2_ passivation layer was deposited by means of PECVD and patterned via wet chemical etching to open the D/S contact holes. Lastly, the TFTs were formed with a channel width/length of 100 μm/20 μm and were thermally annealed in air ambient at T = 300 °C for 1 h. In addition, to measure the band offset of the ITO/a-IGZO heterointerface and the ITON/a-IGZO heterointerface, samples including a 50 nm thick a-IGZO film grown on a Corning 1737 glass substrate, a 50 nm thick ITO film grown on a Corning 1737 glass substrate, a 50 nm thick ITON film grown on a Corning 1737 glass substrate, a 4 nm thick a-IGZO film grown on a 50 nm thick ITO (ITO/a-IGZO heterojunction), and a 4 nm thick a-IGZO film grown on a 50 nm thick ITON (ITON/a-IGZO heterojunction) were characterized via X-ray photoelectron spectrometry (ULVAC-PHI from Takasaki City in Japan, PHI 5000 VersaProbe) with a monochromatic Al Kα (1486.68 eV) X-ray source. The spectra in this study were calibrated using the absorbed C1s at a peak of 284.6 eV. The films were fabricated under the same conditions as the devices.

To analyze the electrical transport mechanism of heterojunction TFTs, technology computer-aided design (TCAD) was applied to simulate a-IGZO TFTs, bilayer ITO/a-IGZO TFTs, and ITON/a-IGZO TFTs. ATLAS and Tony plot tools of the TCAD simulation were used for the carrier transport and band structure under the TFTs’ operation state considering channel material properties such as electron affinity, optical bandgap, and band offset. Meanwhile, the density of states (DOS) model was used independently for both the a-IGZO and ITO layers by fitting the measured experimental data of the TFTs. The DOS in the bandgap of a-IGZO was modeled using four bands of acceptor-like tail states (*g_TA_*(*E*)), acceptor-like deep-level states (*g_GA_*(*E*)), donor-like tail states (*g_TD_*(*E*)), and donor-like deep-level states (*g_GD_*(*E*)). The DOS in the bandgap of the ITO was modeled using two bands of *g_TA_*(*E*) and *g_TD_*(*E*) [22]. The tail states were expressed by an exponential function, and the deep-level states were described by a Gaussian distribution. The specific mathematical models were as follows [23]:

(1) $$g_{TA}\;\left(E\right)=N_{TA}\left(E\right)exp\left(\frac{E-E_{C}}{W_{TA}}\right)$$

(2) $$g_{TD}\;\left(E\right)=N_{TD}\left(E\right)exp\left(\frac{E_{V}-E}{W_{TD}}\right)$$

(3) $$g_{GA}\;\left(E\right)=N_{GA}\left(E\right)exp\left[{-\left(\frac{E_{GA}-E}{W_{GA\;}}\right)}^{2}\right]$$

(4) $$g_{GD}\;\left(E\right)=N_{GD}\left(E\right)exp\left[{-\left(\frac{E-E_{GD}}{W_{GD\;}}\right)}^{2}\right]$$

where *N_TA_* and *N_TD_* are the effective density at the conduction band minimum (*E_C_*) and valence band maximum (*E_V_*), respectively. *W_TD_* and *W_TA_* are the characteristic slope energy of the tail states. *N_GD_* and *N_GA_* are the total density of the Gaussian donor and acceptor states, respectively. *E_GA_* and *E_GD_* are the corresponding peak energies. *W_GA_* and *W_GD_* are the corresponding characteristic decay energies.

## 3. Results and Discussions

Figure 1c shows the transfer curves of a-IGZO TFTs and ITO/a-IGZO heterojunction TFTs with different ITO thicknesses (*t_ITO_*). The detailed electrical parameters of the devices, including threshold voltage (*V_th_*), field effect mobility (*µ_FE_*), subthreshold swing (*SS*), and on/off current ratio (*I*_on/off_), are summarized in Table 1. The threshold voltage of TFTs is determined by linearly fitting the plot of the square root of drain current (*I_DS_*) versus gate voltage (*V_GS_*) by using the following equation:

(5) $${\;I}_{DS}=\frac{W\mu_{FE}C_{ox}}{2L}{\left(V_{GS}-V_{th}\right)}^{2}$$

where *W* is the channel width, *L* is the channel length, and *C_ox_* is the capacitance per unit area of the gate dielectric. The *µ_FE_* in the saturation region (*V_DS_* = 10 V) is extracted by means of the normal method of the square root *I_DS_* versus *V_GS_* plot, and the *SS* is calculated via the inverse of the maximum slope of transfer curves. It is found that the ITO/a-IGZO heterojunction TFTs are transformed to the depletion mode (*V_th_* = −4.9 V) when *t_ITO_* increases to 5 nm, which is due to the high carrier concentration in the bottom ITO channel. As *t_ITO_* increases to 7 nm, the *I_DS_* of the TFTs is weakly modulated by *V_GS_*. Thus, these devices are excluded in subsequent discussions. The detailed electrical parameters are listed in Table 1. It is clear that the electrical properties of bilayer ITO/a-IGZO heterojunction TFTs with a *t_ITO_* of 3 nm are apparently improved compared to those of a-IGZO TFTs. For example, the *µ_FE_* is increased from 6.8 cm^2^ V^−1^s^−1^ to 12.4 cm^2^ V^−1^s^−1^, and the *V_th_* is reduced from 4.8 V to 2.8 V. This result may be caused by the increased carrier concentration in the total channels [17,24,25]. In contrast, the *µ_FE_* of bilayer ITON/a-IGZO heterojunction TFTs is decreased when nitrogen is doped into the ITO thin film, which indicates that the carrier concentration is decreased by N doping [26,27].

**Figure 2:**
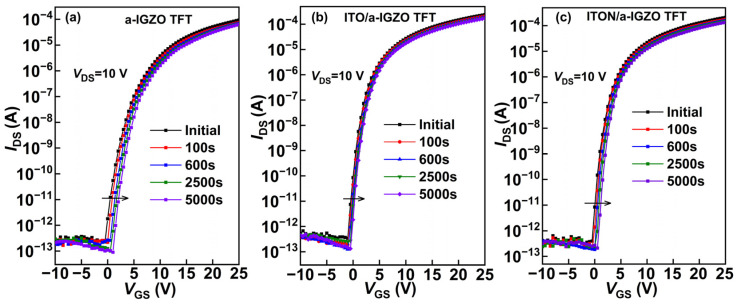
Evolution of the transfer curves as a function of PBS time for (**a**) a-IGZO TFTs; (**b**) ITO/a-IGZO heterojunction TFTs; (**c**) ITON/a-IGZO heterojunction TFTs.

The electrical stability of a-IGZO TFTs, ITO/a-IGZO TFTs and ITON/a-IGZO TFTs was tested under positive gate bias stress (PBS). The tested TFTs were stressed at *V*_GS_ = 20 V for 5000 s. Figure 2a–c show the evolution of transfer curves as a function of PBS duration for the a-IGZO TFTs, ITO/a-IGZO TFTs and ITON/a-IGZO TFTs, respectively. For the stressed TFTs, the transfer curves of the devices parallel shift in the positive direction, and there is a little degradation of their *µ_FE_* and *SS*. This result agrees with previous reports indicating that moderate PBS does not considerably generate additional trap states within a-IGZO TFTs [28]. As a result, the positive threshold voltage drift (Δ*V*_th_) should be attributed to the field-induced electron trapping at the channel/gate dielectric interface [29]. It is clearly observed that the ITO/a-IGZO heterojunction TFTs apparently exhibit better electrical stability compared with the a-IGZO TFTs and ITON/a-IGZO heterojunction TFTs after PBS. Correspondingly, the Δ*V*_th_ of the ITO/a-IGZO heterojunction TFTs (0.5 V) is lower than that of the a-IGZO TFTs (2.1 V) and ITON/a-IGZO heterojunction TFTs (0.7 V). The improved bias stability of bilayer ITO/a-IGZO heterojunction TFTs could be attributed to the suppression of the electron trapping phenomenon by the formation of an energy barrier between the ITO and a-IGZO channel layers [20,29].

To reveal the mechanism of the enhanced electrical performance of ITO/a-IGZO heterojunction TFTs, the energy band alignment of the ITO/a-IGZO heterointerface is analyzed using XPS and optical transmittance. In this work, the valence band offset (∆*E_V_*) at the heterointerface of ITO/a-IGZO is calculated by using Kraut’s method based on the core-level (CL) photoelectric emission. The valence band maximum (VBM) of the samples is defined by using a linear extrapolation method. To determine the ∆*E_V_*, the binding energy differences between the VBM and the selected core peaks for the single layers are combined with the CL binding energy differences for the heterojunction samples. Based on Kraut’s model, the ∆*E_V_* of ITO/a-IGZO heterojunction interface can be described by the following equation [30,31]:

(6) $$∆E_{V}=\left(E_{CL}^{ITO}-E_{VBM}^{ITO}\right)-\left(E_{CL}^{IGZO}-E_{VBM}^{IGZO}\right)-(E_{CL}^{ITO}-E_{CL}^{IGZO})$$

where $E_{CL}^{ITO}$ and $E_{VBM}^{ITO}$ are the binding energy of the selected CL peak and the valence band maximum (VBM) of the bulk ITO film, respectively; $E_{CL}^{IGZO}$ and $E_{VBM}^{IGZO}$ are the binding energy of the selected CL peak and the VBM of the bulk a-IGZO film, respectively; and $(E_{CL}^{ITO}$ − $E_{CL}^{IGZO})$ corresponds to the CL peak difference of the ITO/a-IGZO heterojunction.

**Figure 3:**
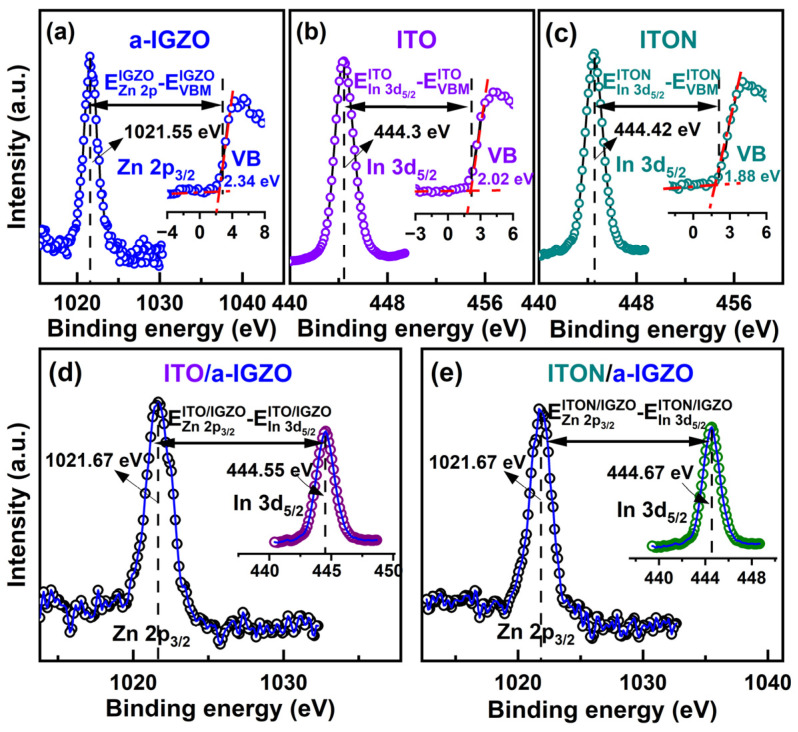
(**a**) Zn 2p_3/2_ XPS and valence band spectra of the a-IGZO film. (**b**,**c**) In 3d_5/2_ XPS and valence band spectra of the ITO and the ITON film, respectively. (**d**,**e**) Zn 2p_3/2_ and In 3d_5/2_ XPS spectra of the ITO/a-IGZO film and ITON/a-IGZO film, respectively.

Figure 3a–c show the CL spectra of Zn 2p_3/2_, In 3d_5/2_ and the VBM (inset) for the a-IGZO film, ITO film and ITON film, respectively. The VBM values of a-IGZO, ITO and ITON are calculated by linearly fitting the leading edge of the *E*_V_ and the flat energy distribution and obtaining the intersection of the two lines [32]. As shown in Figure 3a, it is found that the Zn 2p_3/2_ of the bulk a-IGZO film is located at 1021.55 eV, and the VBM value of 2.34 eV is deduced from the VB spectra via linear fitting as described above. Thus, the energy difference between the Zn 2p_3/2_ CL peak and the VBM of the bulk a-IGZO film is determined to be 1019.21 eV. The CL spectrum of In 3d_5/2_ and the VBM for the bulk ITO film are shown in Figure 3b. The In 3d_5/2_ is located at 444.3 eV, and the VBM value of 2.02 eV is deduced from the VB spectra for the bulk ITO film. As a result, the energy difference between the In 3d_5/2_ CL peak and the VBM is calculated to be 442.28 eV for bulk ITO film. As shown in Figure 3c, the In 3d_5/2_ is located at 444.42 eV, and the VBM value of 1.88 eV is deduced from the VB spectra for bulk ITON film. Therefore, the energy difference between the In 3d_5/2_ CL peak and the VBM is calculated to be 442.54 eV for the bulk ITON film. Furthermore, the CL spectra of Zn 2p_3/2_ and In 3d_5/2_ (inset) of the ITO/a-IGZO and ITON/a-IGZO heterojunction are shown in Figure 3d,e. Compared with the spectra of the bulk a-IGZO film, bulk ITO film and bulk ITON film, the Zn 2p_3/2_ CL peak and the In 3d_5/2_ CL peak in the ITO/a-IGZO and ITON/a-IGZO heterojunction interfaces are shifted to 1021.67 eV, 444.55 eV, and 444.67 eV, respectively. The Zn 2p_3/2_ CL peak position difference between the bulk a-IGZO and the ITO(N)/a-IGZO heterojunction may be due to the chemical bonding of a-IGZO with ITO(N) at the heterojunction interface. The corresponding parameters extracted from XPS measurements are summarized in Table 2. Based on Equation (6), the values of Δ*E_V_* for the ITO/a-IGZO and ITON/a-IGZO interfaces are determined to be 0.19 eV and 0.33 eV, respectively.

**Figure 4:**
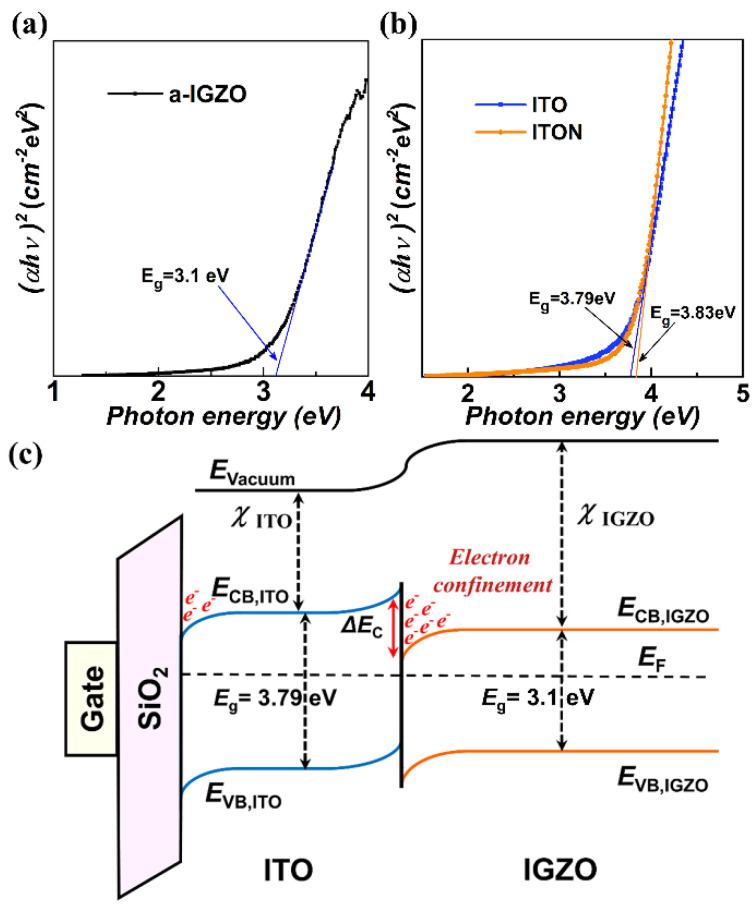
The calculated band gap energies for (**a**) a-IGZO films and (**b**) ITO and ITON films deposited on glass substrates. (**c**) Schematic diagram of the calculated band structure of the ITO/a-IGZO heterointerface.

To calculate the Δ*E_C_* values of the ITO/a-IGZO and ITON/a-IGZO heterostructures, the optical band gap (*E*_g_) of the a-IGZO film, ITO film and ITON film grown on glass substrates are determined by sharply increasing the absorption region based on the Tauc law [33]. Figure 4a,b show the plot of (αhv)^2^ versus hv, where α, h, and v are the absorbance, Planck constant, and light frequency, respectively. The estimated *E*_g_ of the a-IGZO film, ITO film and ITON film are 3.1 eV, 3.79 eV and 3.83 eV, respectively, agreeing with previous reports [34,35,36]. Band-gap widening in N-doped ITO thin films can be ascribed to the reduction in the crystal lattice constant [26,37]. The ∆*E_C_* of the ITO/a-IGZO and ITON/a-IGZO heterojunction interfaces can be estimated based on the following expression: $∆E_{C}=E_{g}^{ITO}-E_{g}^{IGZO}-∆E_{V}$. Based on this expression, the Δ*E_C_* is deduced to be 0.5 eV and 0.4 eV for the ITO/a-IGZO and ITON/a-IGZO heterojunction interface, respectively. The comparatively large Δ*E_C_* will form an architecture containing both a potential well and barrier at the heterojunction interface, which is helpful to accumulate carrier and suppress the electron trapping phenomenon for ITO/a-IGZO heterojunction TFTs. As a result, the calculated Δ*E_C_* values are consistent with the results indicating that the ITO/a-IGZO TFTs exhibit a higher *µ_FE_* and bias stress stability than the ITON/a-IGZO TFTs after PBS. In addition, based on the calculated values, the energy band alignment of the ITO/a-IGZO heterojunction is shown in Figure 4c.

**Figure 5:**
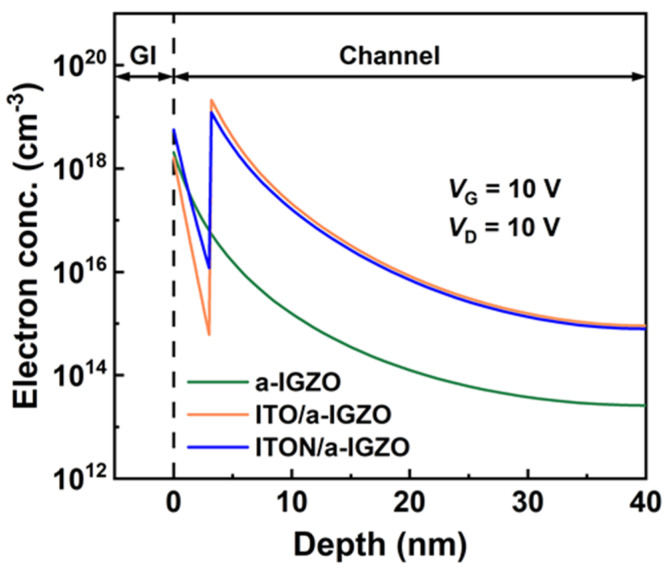
Simulation of the electron concentration distribution of a-IGZO TFTs, ITO/a-IGZO TFTs and ITON/a-IGZO TFTs.

To further confirm the conduction properties of the ITO/a-IGZO heterojunction TFTs, the TCAD simulation of the band structure of a-IGZO TFTs, ITO/a-IGZO heterojunction TFTs, and ITON/a-IGZO heterojunction TFTs is next carried out to quantitatively analyze the devices’ transport behavior. In the simulation, the model experimental parameters are incorporated from the fabricated devices, such as the carrier mobility, optical bandgap, band offset, and permittivity. Meanwhile, the DOS model is applied independently for the a-IGZO, ITO, and ITON channel layers by fitting the measured experimental data of the TFTs. The parameters used in the TCAD simulation for a-IGZO TFTs, ITO/a-IGZO heterojunction TFTs, and ITON/a-IGZO heterojunction TFTs are summarized in Table 3. As shown in Figure 5, the carrier concentration distribution of a-IGZO TFTs, ITO/a-IGZO heterojunction TFTs, and ITON/a-IGZO heterojunction TFTs at *V*_GS_ = 10 V is extracted. Compared with a-IGZO TFTs, it is found that there is a high density of carriers (~2.1 × 10^19^ cm^−3^) distributed at the ITO/a-IGZO interface for the ITO/a-IGZO heterojunction TFTs. The distribution of carriers at the ITO/a-IGZO interface is similar to that in a q2DEG system at the heterointerface [38,39], which is beneficial for decreasing carrier scattering and enhancing the µ_FE_ of heterojunction TFTs. This result agrees with the improved µ_FE_ for the ITO/a-IGZO heterojunction TFTs, which can be attributed to the increased carrier concentration in the channel. In contrast, it is found that the distributed electron concentration (~1.4 × 10^19^ cm^−3^) at the ITON/a-IGZO interface is less than that of the ITO/a-IGZO interface, which can be attributed to the decrease in the number of electrons in the N-doped ITO channel layer [12,40,41]. Correspondingly, the µ_FE_ of the ITON/a-IGZO heterojunction TFTs is less than that of the ITO/a-IGZO heterojunction TFTs due to the decrease in carrier accumulation at the device heterojunction interface. As a result, the distributed carrier concentration at the ITO/a-IGZO interface plays an important role in modulating the electrical performance of heterojunction TFTs.

## 4. Conclusions

In this work, bilayer ITO/a-IGZO heterojunction TFTs are designed based on the strategy of bandgap engineering. Compared to a-IGZO TFTs, the mobility (*µ_FE_*) of the ITO/a-IGZO heterojunction TFTs is increased from 6.8 cm^2^ V^−1^s^−1^ to 12.4 cm^2^ V^−1^s^−1^, and the Δ*V*_th_ of the ITO/a-IGZO heterojunction TFTs is decreased from 2.1 V to 0.5 V. By means of XPS characterization, the ∆*E_C_* of ITO/a-IGZO and ITON/a-IGZO heterojunctions has been determined to be 0.5 eV and 0.4 eV, respectively. The results indicate that the formed potential well at the ITO/a-IGZO heterointerface can cause electronic accumulation, which is beneficial for improving the *µ_FE_* of the ITO/a-IGZO TFTs. Meanwhile, the comparatively large Δ*E_C_* can effectively reduce the electron trapping phenomenon and improve the bias stress stability of heterojunction TFTs. Moreover, it is found that there is a high density of carriers distributed at the ITO/a-IGZO interface for the heterojunction TFTs via the TCAD simulation, which indicates that the distributed carrier concentration at the heterojunction interface plays a key role in determining the *µ_FE_* of the heterojunction TFTs. As a result, the heterojunction channel structure will provide an approach to overcome the trade-off between *µ_FE_* and bias stability in a-IGZO TFTs.

## Figures and Tables

**Table 1:** Extracted electrical parameters of the a-IGZO TFTs, ITON/a-IGZO TFTs and ITO/a-IGZO TFTs with different ITO thicknesses.

Channel Structure	*V*_th_ (V)	*μ_FE_* (cm^2^ V^−1^s^−1^)	*SS* (V dec^−1^)	*I*_on/off_
a-IGZO ITO/a-IGZO (ITO 3 nm)	4.8 2.8	6.8 12.4	0.7 0.46	>10^8^ >10^8^
ITO/a-IGZO (ITO 5 nm)	−4.9	13.2	0.55	>10^8^
ITON/a-IGZO (ITON 3 nm)	3.2	11.2	0.41	>10^8^

**Table 2:** The binding energies (eV) of the XPS peaks and VBM for the a-IGZO, ITO, ITON, ITO/a-IGZO and ITON/a-IGZO samples.

Sample	Metal E_CL_	Peak (Bulk)	Peak (Interface)	Peak Shift	VBM
a-IGZO	Zn 2p_3/2_	1021.55	1021.67	0.12	2.34
ITO	In 3d_5/2_	444.3	444.55	0.25	2.02
ITON	In 3d_5/2_	444.42	444.67	0.25	1.88

**Table 3:** The parameters used in the TCAD simulation for a-IGZO, ITO/a-IGZO and ITON/a-IGZO TFTs.

Parameters	a-IGZO	ITO	ITON	Description
*N_TA_* (eV^−1^cm^−3^)	2.45 × 10^20^	1 × 10^20^	2.5 × 10^20^	Accept-like tail states at *E*_C_
*W_TA_* (eV)	0.05	0.02	0.1	Conduction-band-tail slope
*N_GA_* (eV^−1^cm^−3^)	3.2 × 10^17^	-	-	Peak of O_i_ states
*W_GA_* (meV)	0.44	-	-	Characteristic decay energy
*E_GA_* (eV)	0.8	-	-	Peak energy
*N_TD_* (eV^−1^cm^−3^)	1 × 10^19^	4 × 10^20^	1.5 × 10^20^	Donor-like tail states at *E*_V_
*W_TD_* (eV)	0.2	0.11	0.11	Valence-band-tail slope
*N_GD_* (eV^−1^cm^−3^)	6 × 10^17^	-	-	Peak of Gaussian donor-like states
*W_GD_* (eV)	0.3	-	-	Characteristic decay energy
*E_GD_* (eV)	2.9	-	-	Peak energy
*E*_g_ (eV)	3.1	3.79	3.83	Optical band gap
Δ*E_C_* (eV)	-	0.5 (ITO/a-IGZO)	0.4 (ITON/a-IGZO)	Conduction band offset

## Data Availability

The original contributions presented in the study are included in the article, further inquiries can be directed to the corresponding author.
